# Could Exogenous Insulin Ameliorate the Metabolic Dysfunction Induced by Glucocorticoids and COVID-19?

**DOI:** 10.3389/fendo.2021.649405

**Published:** 2021-06-18

**Authors:** Martin Brunel Whyte, Prashanth R. J. Vas, Anne M. Umpleby

**Affiliations:** ^1^ Faculty of Health Sciences, University of Surrey, Guildford, United Kingdom; ^2^ King’s College Hospital NHS Foundation Trust, London, United Kingdom

**Keywords:** coronavirus – COVID-19, insulin, glucocorticoid, critical-illness, dexamethasone

## Abstract

The finding that high-dose dexamethasone improves survival in those requiring critical care due to COVID-19 will mean much greater usage of glucocorticoids in the subsequent waves of coronavirus infection. Furthermore, the consistent finding of adverse outcomes from COVID-19 in individuals with obesity, hypertension and diabetes has focussed attention on the metabolic dysfunction that may arise with critical illness. The SARS coronavirus itself may promote relative insulin deficiency, ketogenesis and hyperglycaemia in susceptible individuals. In conjunction with prolonged critical care, these components will promote a catabolic state. Insulin infusion is the mainstay of therapy for treatment of hyperglycaemia in acute illness but what is the effect of insulin on the admixture of glucocorticoids and COVID-19? This article reviews the evidence for the effect of insulin on clinical outcomes and intermediary metabolism in critical illness.

## Trials of Glucocorticoids in COVID-19

### The RECOVERY Trial

At the time of writing, no pharmacological intervention for COVID-19 has been as successful as steroids for treating the acute illness. The Randomized Evaluation of COVID-19 Therapy (RECOVERY) trial showed that dexamethasone 6mg daily for 10 days reduced the mortality of mechanically ventilated patients by 29% (1). This was despite 8% of the usual care group receiving Dexamethasone in RECOVERY, which would bias results towards the null, raising the possibility of even greater benefit. However, mortality was measured at 28 days and longer-term data will be informative as the adverse impacts of steroid administration (in other acute conditions) may be seen up to 90 days (2).

i) Dose: It is not entirely clear how the dose of 6 mg was decided upon. Immediately prior to the pandemic, the Dexamethasone treatment for the acute respiratory distress syndrome: a multicentre, randomised controlled trial’ (DEXA-ARDS) reported on outcomes in ARDS of dexamethasone at a starting dose of 20mg daily (3). This dose is consistent with prior studies of ARDS (which used methylprednisolone regimens dosed at 1-2 mg/kg/day initially) (4).

A common pattern evolving from five retrospective trials early in the course of the pandemic was for greater benefit with low dose steroids compared to the high dose steroids (5). It is likely that the 6mg dose was a trade-off between the beneficial effects of resolving pulmonary and systemic inflammation and supporting blood pressure; and the adverse effects of inhibiting immune response, reduce pathogen clearance, and provoking viral replication (6).

ii) Pharmacokinetics: In RECOVERY, the trial drug could be given orally or intravenously and surprisingly, the route of administration was not recorded in the study documentation. It is conceivable that for patients receiving mechanical ventilation, the route of administration was more likely to be intravenous, whereas it was probably given orally outside of this subgroup. The bioavailability of oral dexamethasone is between 70% and 78%, and therefore dexamethasone in tablet form may not have an equivalent therapeutic effect (7).

### Meta-Analysis of Glucocorticoids in COVID-19

Following release of RECOVERY outcomes, several ongoing hydrocortisone trials were stopped as it was considered ethically imperative to use dexamethasone. This reduced the numbers of participants and hard end points were not achieved.

A meta-analysis was undertaken by WHO Rapid Evidence Appraisal for COVID-19 Therapies (REACT) Working Group (8). This incorporated data from seven trials (RECOVERY, REMAP-CAP, CoDEX, CAPE COVID, and three additional trials) totalling 1703 patients (678 had been randomized to corticosteroids and 1025 to usual care or placebo), hospitalized with COVID-19 critical illness.

The 28-day mortality was lower in patients randomised to corticosteroids: 222 deaths among 678 patients randomized to corticosteroids compared with 425 deaths among 1025 patients randomised to usual care or placebo (odds ratio [OR], 0.66 [95% CI, 0.53-0.82]; P < 0.001). The RECOVERY trial provided 59% of the patients (8). In the analysis that excluded patients recruited to the RECOVERY trial, the OR was 0.77 (95% CI, 0.56-1.07) for all-cause mortality comparing corticosteroids with usual care or placebo. The point-estimate for reduced mortality was similar between dexamethasone and hydrocortisone: OR for mortality reduction was 0.64 (95% CI 0.50 to 0.82) with dexamethasone and 0.69 (0.43 to 1.12; P=0.13) with hydrocortisone). Of note, the only trial that assessed methylprednisolone (Steroids-SARI) was underpowered and OR for effect was 0.91 with wide confidence interval (0.29 to 2.87). Outcomes were also similar with lower- *vs* higher-dose corticosteroid regimens (demarcation between low and high-dose was pre-specified at 15 mg/d of dexamethasone, 400 mg/d of hydrocortisone, and 1 mg/kg/d of methylprednisolone).

Since publication of the WHO meta-analysis, a more recent randomised, placebo-controlled, double-blind study of 0.5 mg/kg of methylprednisolone conducted in Brazil in 393 patients found no difference in 28-day mortality and patients on steroids required more insulin therapy (9). However, the Brazilian cohort were on average about ten years younger than in RECOVERY, had less heart disease (7% *vs* 28%), and there was a greater proportion on mechanical ventilation at enrolment – suggesting more severe disease (33.8% on mechanical ventilation *vs* 15.5% without). This runs counter to the idea that greater benefit is seen in more severely unwell patients - the majority of the studies in the WHO meta-analysis were conducted in patients with serious or critically unwell patients, particularly those who required high flow nasal oxygen or ventilation (8). In the RECOVERY trial itself, there was no benefit among those who were receiving no respiratory support at randomization (17.8% dexamethasone *vs*. 14.0% control; rate ratio, 1.19; 95% CI, 0.91-1.55).

## Implications of COVID-19, and the RECOVERY Trial Protocol, on Diabetes

Diabetes was present in 24% of dexamethasone group *vs* 22% of usual care of the RECOVERY trial. The study investigators did not adjust for multiplicity in the study, between treatment arms or for any of the pre-specified endpoints, meaning there is a potential inflation of the type I error rate. This would be more of an issue for some of the secondary endpoints. Even so, it is surprising that data for patients with diabetes was not reported. Two serious adverse events (SAEs) for hyperglycaemia, requiring a longer admission for stabilisation, were recorded in the dexamethasone group (10). Six milligrams of dexamethasone OD is, in effect, five‐ to six-fold greater than the therapeutic glucocorticoid replacement dose and therefore metabolic perturbation is to be anticipated but the extent of this is uncertain.

Prior to the COVID-19 pandemic, few papers examined the acute effects of steroids on glucose homeostasis, when newly administered to general medical inpatients. In these studies (11–14), up to 50–70% of hospitalized patients (without known diabetes) prescribed moderate-to-high glucocorticoid doses, developed hyperglycaemia.

New hyperglycaemia (capillary glucose ≥11 mmol/L after initiation of glucocorticoid therapy) was found in 14% of general medical admissions treated with the equivalent of 30mg prednisolone (~4.5mg of dexamethasone daily), over a short period of time (median 2.5 days; interquartile range [IQR] 1-4 days) (11). At higher doses of prednisolone (~40mg daily) over four weeks - and including a subgroup receiving pulsed methylprednisolone 500-1000mg per day - two-thirds of patients developed steroid-induced diabetes (14). In these studies of individuals without diabetes, SID was more likely with older age, higher HbA_1c_ level, lower estimated glomerular filtration rate (eGFR) and greater illness severity (11–14).

In a meta-analysis of two randomised controlled trials, single-dose 8mg dexamethasone, administered pre-operatively, led to a mean 0.39mmol/L higher blood glucose than control, after 24 hours (95% CI: 0.04 - 0.74 mmol/L, P=0.03) (15–17). Extended data, past 24 hours, is unavailable. Given the long half-life of dexamethasone (36-54 hours), a prolonged effect might be anticipated. Continuous day-curves of glucose sampling after dexamethasone are not reported but after a single pre-operative dose 10mg dexamethasone in people without diabetes, peak glucose was 2.5 mmol/L higher at 4 hours compared to control (18), and significant increment, within 2 hours may be seen after intravenous dosing (19).

## COVID-Specific Effects on Glucose Handling

There may be a bidirectional relationship between diabetes and COVID-19 whereby COVID-19 can worsen, or precipitate diabetes and the presence of diabetes may worsen the severity of the COVID-19 illness (20). A positive feedback loop is thus engendered.

### COVID-19 Causing Hyperglycaemia and Diabetic Ketoacidosis

Acute hyperglycaemia has been seen in individuals infected with SARS-CoV-2 but without known diabetes (21–26). In these patients, the degree of admission hyperglycemia predicts mortality and disease severity. The risk of COVID-19-related hospitalisation and mortality has also been shown to be greater in individuals with long-term hyperglycaemia (represented by higher HbA_1c_) (27–29).

Hyperglycaemia in COVID-19 may represent an effect on insulin resistance but it has also been questioned whether insulin production might also be affected. A decade ago, it was hypothesized that SARS coronavirus may directly damage islet cells (30). More recently, *in vitro* studies suggest that SARS-CoV-2 infection of pancreatic endocrine cells results in robust chemokine induction and upregulation of markers of cell death (31). Observational data of clinical outcomes provides support for a direct pancreatic insult: diabetic ketoacidosis (DKA) has been associated with COVID-19 disease (32–34). Reports from China, early in the pandemic, suggested ketosis was a relatively frequent occurrence: of 658 patients, 42 (6.4%) presented with positive urine or serum ketones, and, of these, three (7%) patients met the American Diabetes Association (ADA) criteria for DKA (33). Those with ketosis were about twice as likely to have diabetes at baseline, and the 3 individuals who developed DKA had underlying diabetes (one with type 1 diabetes, two with type 2 diabetes). A marked increase in DKA was also observed in children and adolescents in Germany and Australia during the COVID-19 pandemic (35, 36). However, other groups have found no increased incidence of new-onset type 1 diabetes during this COVID-19 pandemic, compared to historical rates (37). Furthermore, antibody positivity to SARS-CoV-2 has not been associated with greater risk of type 1 diabetes in children (38).

SARS-CoV-2 enters human cells *via* co-expression of its cell entry factors, angiotensin-converting enzyme 2 (ACE2) and its obligate co-factor, transmembrane serine protease 2 (TMPRSS2). However, analysis of six transcriptional datasets of primary human islet cells found that *ACE2* and *TMPRSS2* were not co-expressed in single β cells (39), suggesting that direct viral entry is not the means of pancreatic damage with COVID-19.

There have been small case series of individuals with COVID-19 with mildly raised lipase and/or amylase but not meeting the criteria for pancreatitis (40). Therefore there is no convincing evidence for more diffuse pancreatic injury as a mechanism for insulinopaenia.

Black individuals have been particularly affected by COVID and are over-represented in series with ketosis (41). It is possible that the clinical picture of ketosis in COVID relates, in part, to unmasking ketosis-prone type 2 diabetes (KPDM) – which has been linked with Black ethnicity (42). Alternatively, increase in the prevalence of severe DKA in COVID-19 positive patients might relate to delayed hospital admission and/or accessing medical advice.

In summary, ketosis is associated with length of hospital admission and overall mortality (33). The data appear to show that Covid-19 causes DKA more often than other respiratory tract viral infections.

### Diabetes Predisposing to Infection With COVID-19

Elevated glucose levels can directly induce SARS-CoV-2 viral replication in human monocytes. Glycolysis appears to sustain SARS-CoV-2 replication *via* the production of mitochondrial reactive oxygen species and activation of hypoxia-inducible factor 1α (HIF1α) (43). HIF1α, in turn, upregulates glycolytic genes and IL-1β expression. Therefore, acute hyperglycaemia might directly support viral proliferation. Furthermore, people with diabetes have a number of pathophysiological changes that may underlie a more severe clinical response to COVID-19, these include: greater proinflammatory cytokine release, compromised host immune responses, endothelial dysfunction, and a greater propensity for development of coagulation-related complications (44, 45). Taken together, diabetes leads to greater viral replication and more severe COVID-19 disease, leading to greater hyperglycaemia.

## Effect of Critical Illness on Glucose, Fat and Protein Metabolism

### Endogenous Hypercortisolaemia

Under non-stressed conditions, the adrenal cortex produces approximately 20 mg of cortisol during the day. This then increases within 4-6 hours of acute stress, from a baseline of approximately 400 nmol/l, to a peak of more than 1500 nmol/L (depending on the severity of illness) (46). Estimates for equivalency of hydrocortisone dosing have ranged from 60 to 200 mg cortisol per day (47, 48).

**Figure 1:**
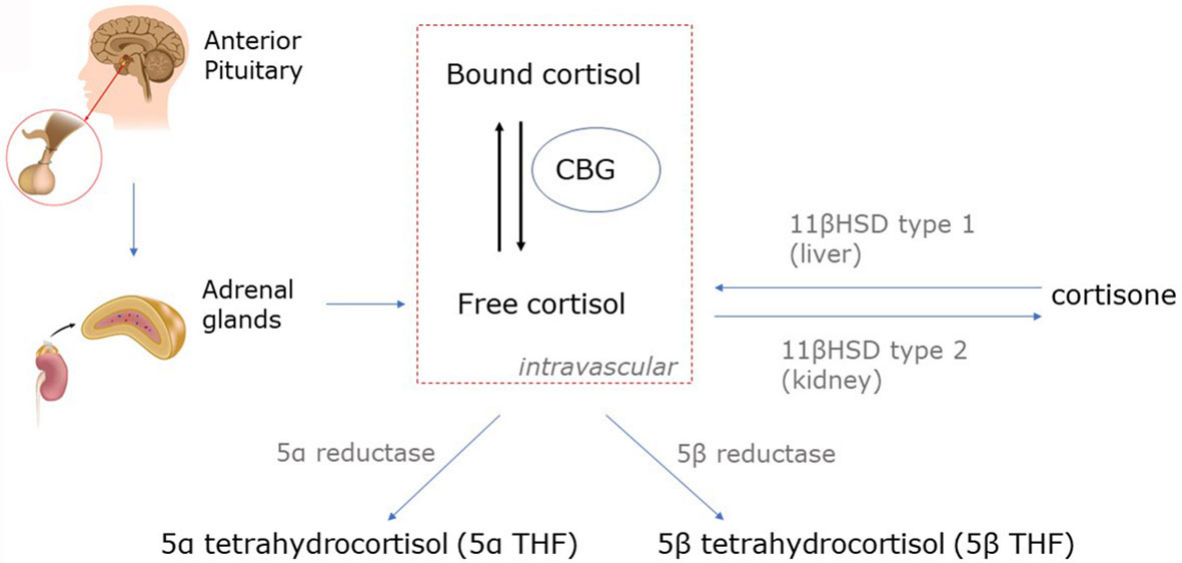
Cortisol metabolism. Cortisol is converted in peripheral tissues to cortisone by 11β-hydroxysteroid dehydrogenase (11β-HSD). Cortisone has marginally reduced glucocorticoid activity compared to cortisol (80-90%), and thus, cortisone can be considered an active metabolite of cortisol. Unbound cortisol is biologically active, but the majority of circulating cortisol is bound to corticosteroid-binding globulin (CBG) and albumin. Cortisol is metabolized by 5α- and 5β reductases to form 5α- and 5β-tetrahydrocortisol (5α- and 5β-THF).

Cortisol production is at least partially ACTH-dependent. There is a stimulatory effect on the hypothalamus by inflammatory mediators such as TNFα and IL-1 for the release of CRH (49). Cytokines can also have an effect downstream on the pituitary; for instance, IL-6 appears to directly stimulate the release of ACTH (49). However, the concept of vastly increased corticosteroid production in critical illness has been challenged. Using stable isotope tracers, the rate of appearance of cortisol, was only 1.8-fold higher in critically ill patients than in healthy matched controls in the presence of low morning plasma ACTH values (47). Therefore impaired cortisol clearance likely also contributes to hypercortisolaemia. Hypoperfusion of cortisol-metabolizing organs could, theoretically, reduce cortisol breakdown but there is evidence for reduced hepatic expression and activity of cortisol-metabolizing enzymes 5α- and 5β-reductase and renal 11β-hydroxysteroid dehydrogenase-2 **(** **Figure 1** **)** (47). Cortisol-binding globulin (CBG) decreases in the context of physiological stress (50). The concentration of CBG being negatively associated with mortality in septic shock (51). The net effect of an elevation in total cortisol and a reduction in cortisol-binding globulin will be to increase free cortisol levels (50). Greater cortisol concentration is associated with increased mortality in COVID-19 (52).

Secretion of cortisol can be driven by factors outside the HPA axis in critical illness (53). This is supported by reduced adrenocorticotropic hormone (ACTH) and the increased irregularity and asynchrony of the ACTH and cortisol time series during critical illness (54). A biphasic response to critical illness has been proposed whereby an initial ACTH-dependent process gives way to later non-ACTH pathway (55). Within the adrenal gland, macrophages, and lymphocytes, physiologically widely infiltrating the adrenal cortex, and adrenocortical, and chromaffin cells produce cytokines, as IL-1, IL-6, TNFα, leukaemia inhibitory factor (LIF), and IL-18 which have a key role in the immune-adreno-cortical communication (56).

### Insulin Resistance

Hypercortisolaemia will increase the rate of hepatic gluconeogenesis and inhibit glucose uptake and utilisation by peripheral tissues (57, 58). Unlike in health, where glucocorticoids promote hepatic glycogen storage, acute illness is characterised by markedly reduced glycogen synthesis (59).

The action of glucocorticoids will be compounded by elevated circulating catecholamines, which can antagonise the actions of insulin by several mechanisms: they can stimulate glucagon by a β-adrenergic effect, increase hepatic glucose production by direct stimulation of glycogenolysis and gluconeogenesis and decrease glucose uptake (60). A β2 receptor mediated increase in lipolysis could also exacerbate insulin resistance through ectopic fat distribution, release of adipokines or promoting macrophage infiltration of adipose tissue (61). Critical illness is associated with markedly elevated levels of glucagon which increases hepatic amino acid catabolism, contributing to the illness-induced hypoaminoacidaemia (62). In COVID-19, the profound viral induced inflammation, in particular IL-6 mediated, will further increase insulin resistance (63). The severity of pneumonitis correlates with the insulin requirement, but there does not appear to be a specific effect of COVID-19 on insulin resistance (64).

### Catabolism Induced by Insulin Resistance

**Figure 2:**
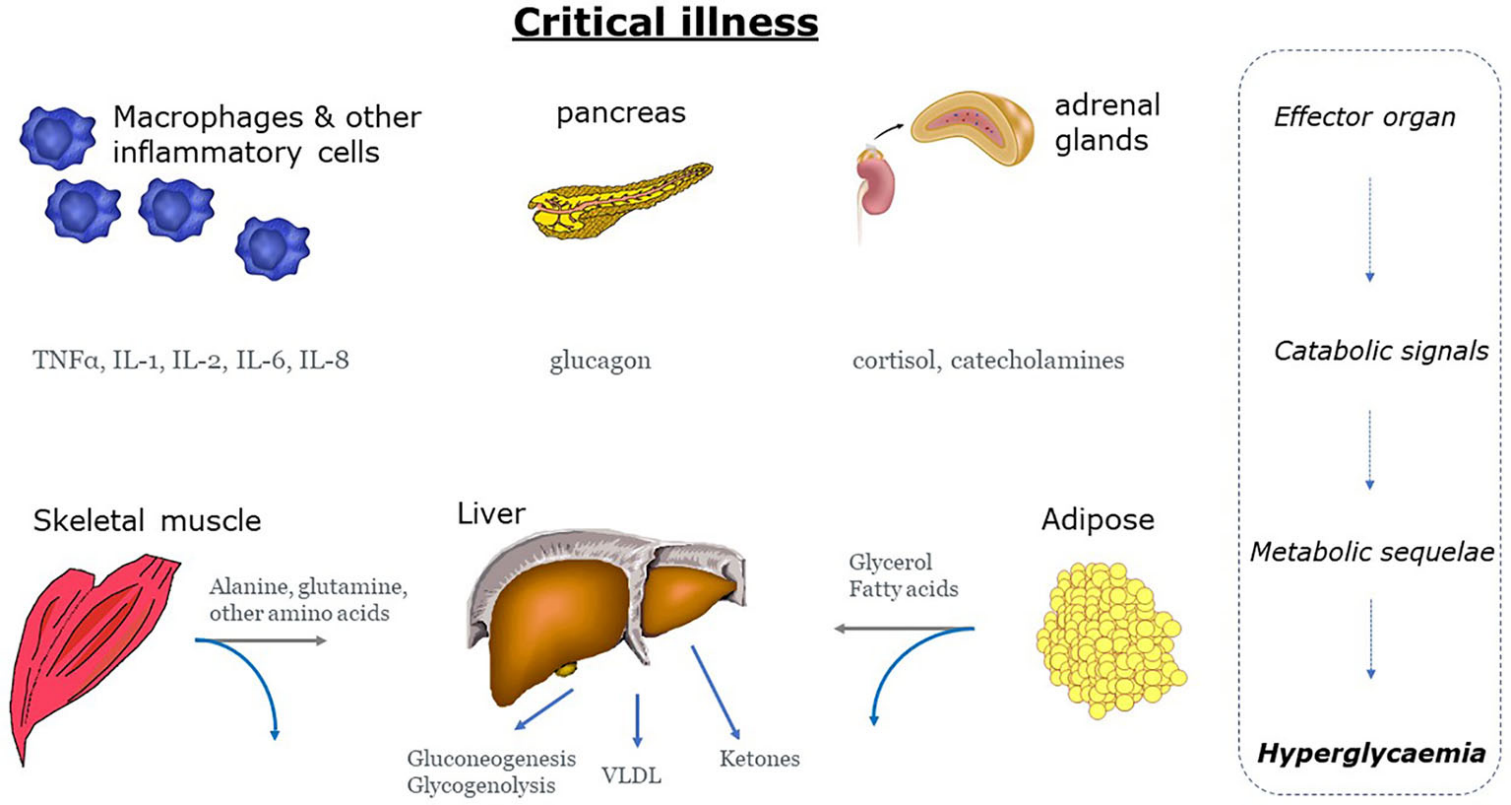
Mechanisms of hyperglycaemia during states of stress and inflammation. Stress hyperglycemia is the end result of a neurohumeral and inflammatory process characterized by excessive gluconeogenesis and glycogenolysis and impaired insulin-mediated glucose uptake. Grey arrows: gluconeogenic precursors IL, interleukin; TNF-α, tumour necrosis factor-α; VLDL, very low-density lipoprotein.

The surge in proinflammatory mediators and counter-regulatory hormones, favours the shift to catabolism marked by insulin resistance - with insulin sensitivity reduced by 70% **(** **Figure 2** **)** (65). Indeed, in the presence of critical-illness, hepatic glucose production increases at least twofold compared to healthy controls, to rates approaching 15 – 25 μmol/kg/min (66, 67). Hyperglycaemia is also the result of diminished insulin-mediated glucose uptake by skeletal muscle (59, 68). Critically ill patients have significantly lower, and more variable insulin sensitivity, on day 1 than later in their intensive care unit (ICU) stay (69, 70), although insulin resistance may persist for months (71). The acute effect is likely due to the acute counter-regulatory response to critical illness as described above. Catabolism, insulin resistance and stress hyperglycaemia are evolutionarily responses designed to allow the host to survive during periods of severe stress. Glucose can be utilized by tissues that are central to the recovery process. These include the central and peripheral nervous system, bone marrow, leucocytes and erythrocytes and the reticuloendothelial system. Glucose uptake to these tissues is non-insulin dependent - hence greater glucose concentration facilitates uptake (72). This evolutionary paradigm - of either survival or rapid deterioration - has been superseded by the ability to ‘suspend’ critical illness for days or weeks with modern critical care. In the modern era, prolonged or severe hyperglycaemia is associated with increased risk of critical illness polyneuropathy and prolonged mechanical ventilation. Loss of lean body mass is associated with poor ICU survival, or delayed recovery in survivors (73).

Metabolomic and lipidomic approaches have shown that circulating triglyceride and fatty acid concentrations correlate with disease severity in COVID-19 (74). This mirrors data from septic patients in the first days of hospital admission (75). Microdialysis catheters have been used in femoral adipose tissue in patients with systemic inflammatory response syndrome/severe sepsis or shock. On day 1 of ICU admission 56% of patients had increased interstitial levels of glycerol and FFA, the two products of lipolysis, with glycerol concentrations being higher in those receiving glucocorticoids (76). Increased very-low density lipoprotein (VLDL) production by the liver also contributes to the elevation of plasma triglyceride concentration in sepsis (77). By contrast, the absorption of lipid from the small intestine is diminished in critical illness (78).

### Protein Catabolism

Negative nitrogen balance has been linked to detrimental clinical outcomes. The survival of critically-ill patients, their duration of ICU admission, and the duration to recovery of normal physiological function, are all inversely correlated with loss of lean body mass during hospitalisation (79). As the largest protein pool, it is unsurprising that the major site of protein loss is from skeletal muscle. Muscle biopsy studies in the critically-ill have shown rapid decreases in myosin heavy-chain mRNA and protein expression by the fifth day of ICU admission (80), with average of 2% loss per day over the first 10 days (80–82). The duration of corticosteroid treatment, independent of duration of intensive care unit stay or other risk factors, is a dominant risk factor for a low myosin/actin ratio (81). Long-term outcomes from ICU-acquired weakness are significant and include lower one-year survival, and reduced walk and exercise ability five-years later (83).

The predominant defect appears to be an accelerated rate of proteolysis that cannot be compensated for by a moderate rise in the rate of protein synthesis (84, 85). There are multiple stimuli for the increase in muscle catabolism, including hormonal and cytokine but regression analysis found that plasma cortisol concentration was the most significant predictor of protein breakdown (where it explained nearly 40% of the variance) (84). These data are consistent with earlier studies in normal subjects, whereby artificial elevation of plasma cortisol - to levels observed after trauma - resulted in a 15% increase in whole body protein breakdown (86). The possibility of hyperglycaemia, itself, acting as a spur for proteolysis has been explored in normal subjects with the use of combined insulin and somatostatin administration (87). Using stable isotopic tracer methodology, hyperglycaemia (~ 10.5mmol/L) was associated with a three-fold increase in proteolysis, without alteration of whole-body protein synthesis or protein oxidation compared to normoglycaemia (~5.2mmol/L). A retrospective review of burned patients suggested a correlation between the extent of proteolysis and prevailing glycaemia, with maximal proteolysis occurring in patients with plasma glucose above 12.8 mmol/L and least catabolism in those with plasma glucose below 8.6 mmol/L (88). However, both hyperglycaemia and protein degradation may have merely represented the disease severity and by extension, the degree of insulin resistance. Sepsis can significantly increase protein catabolism and exacerbate muscle protein loss in already hypercatabolic patients (89), suggesting a significant role for cytokines as catabolic factors. Cytokines and stress hormones increase protein turnover *via* a common mechanism involving the activation of muscle-specific ubiquitin-ligases (82).

### Proteolysis and Secondary Infection

A catabolic state may compromise the immune response by mechanisms such as poor wound healing, altered mucosal barrier, tissue oedema due to low albumin and reduced muscle strength (and vital capacity) leading to pneumonia. Loss of respiratory muscular power will prolong ventilation and adversely affect the patient’s ability to clear the airways with sufficient cough and thus increase the risk of pneumonia (79). Skeletal muscle contributes in a bidirectional role in systemic inflammatory signalling and the modulation of the inflammatory response including by release of heat shock proteins (HSP) (90). Skeletal muscle provides a key nutrient to the immune system in the form of glutamine (91), which is a constitutively essential amino acid during catabolic situations. Glutamine acts as an energy substrate for leucocytes and is necessary for tissue repair and intracellular pathways associated with pathogen recognition (92). Deficiency of a skeletal muscle amino acid reservoir would render a patient more susceptible to death from multiple organ failure following a ‘second-hit’ episode of sepsis as there would be inadequate substrate supply for immune function.

The proportion of patients with COVID-19 plus secondary bacterial infections ranges from 5% to 30% (93) and the incidence rate of bacterial blood-stream infections among patients with COVID-19 admitted to the ICU appears to be higher than in historical cohorts (93, 94). Rates of bacterial secondary infection in severe COVID-19 will be skewed by prescription of antibiotics - to cover for bacterial superinfection (as with during influenza pandemics) – as advocated by several guidelines (95).

## The Metabolic Effects of a Short-Course of Glucocorticoids

Administration of even relatively low doses of prednisolone (6-7.5 mg daily) over one to two weeks acutely increases basal hepatic glucose production and reduces insulin mediated suppression of hepatic glucose production and stimulation of peripheral glucose disposal (57, 58). Glucocorticoids will inhibit the conversion of pyruvic acid to acetyl-coenzyme A, leading to an accumulation of pyruvic acid and resulting in glucose re-synthesis (96). Induction of gluconeogenic enzymes, such as glucose-6-phosphatase, fructose-1,6-bisphosphatase and phosphoenolpyruvate carboxykinase, add to this effect (97). In the liver, glucocorticoids increase glycogen storage, which can be observed from three to twenty-four hours after the administration of glucocorticoids (96), whereas in skeletal muscle they play a permissive role for catecholamine-induced glycogenolysis and/or inhibit insulin-stimulated glycogen synthesis (98). A negative effect on first- and second-phase insulin release is also seen with glucocorticoids, possibly mediated *via* a reduced insulinotropic effect of glucagon-like peptide-1 (GLP-1) (99, 100).

Acutely, over 5-7 days, glucocorticoids in therapeutic doses can induce protein catabolism, in healthy subjects, by increasing the rate of protein degradation by the ubiquitin-proteasome system and autophagy lysosome system (101) and by increasing whole-body protein oxidation (102). Protein synthesis is also suppressed at the level of translational initiation, preventing the production of new myofibrillar protein (101). A dose-response gradient with worsening whole body protein metabolism at increased steroid doses, has been measured with isotopic techniques (103).

## Anticipated Outcomes of Insulin Use in Hospitalised Patients Receiving Dexamethasone

### Clinical Outcomes

The historic paradigm that hyperglycaemia in critically-ill patients was an adaptive response that provided glucose for the brain, red cells, and wound healing meant that the approach to treatment was to treat the blood glucose only once high enough to cause an osmotic drag and produce a diuresis (approximately 11-12mmol/L). This approach was reconsidered following the publication of two randomised controlled trials from Leuven of insulin use in critically-ill patients (104, 105). The first study involved adults admitted to a surgical ICU with glucose targets in the intervention group of 4.5 - 6.1 mmol/L, compared with a comparatively high ceiling for the control group of 10.0 – 11.mmol/L) (105). Tight control reduced ICU mortality from 8% to 4.6%. Only 13% of the patients had diabetes. Most benefit was amongst patients with multiple organ failure and sepsis. Of importance, 62% of admissions were due to cardiac surgery and an effect of glucose/insulin on the myocardium was postulated. The insulin infusion rate was (mean) 0.04 iU/kg/hr; consuming 9g glucose/hr (105). In contrast, studies using a fixed glucose-insulin-potassium (GIK) regime, with acute myocardial infarction, to promote a switch away from myocardial fatty acid metabolism to glucose metabolism, were approximately 0.1 - 1 iU/kg/hr; 30 - 80 g glucose/hr) (106). Expectation that cardio-metabolic modulation with high-dose insulin could improve outcomes were diminished after the neutral results seen in the large Clinical Trial of Reviparin and Metabolic Modulation in Acute Myocardial Infarction Treatment and Evaluation-Estudios Clinicos Latino America (CREATE-ECLA) (107). Furthermore, *post-hoc* analysis of the Leuven surgical study (105) suggested that the benefit accrued from normoglycaemia, rather than from hyperinsulinaemia (108).

The second Leuven study was in medical ICU patients, where no mortality benefit was seen, except in those requiring ICU stays of three or more days (104). These data suggest that insulin may protect against the development of organ failure (particularly from sepsis), rather than reversing pathological processes once established. Three other studies also did not show benefit in mixed medical and surgical ICUs. The Volume Substitution and Insulin Therapy in Severe Sepsis (VISEP) study enrolled 480 severe sepsis patients who were randomized to tight glycaemic control or standard glucose control (109). VISEP was suspended early for increased rates of hypoglycaemia in the intensive control arm (17.6% *vs* 4.5%) and no difference in 28-day or 90-day mortality. The Glucontrol study was also suspended after enrolment of 1101 patients due to safety and protocol concerns (110). There was no difference in mortality, but rates of hypoglycaemia were approximately 4 times higher in the intensive insulin group (9.8% *vs* 2.7%). The Normoglycemia in Intensive Care Evaluation–Survival Using Glucose Algorithm Regulation (NICE-SUGAR) study randomised 6104 patients to a target of 4.5 - 6.0 mmol/L or to < 10 mmol/L (111). There was a greater risk of mortality in the intensive glycaemic control group (odds-ratio 1.14) with no difference in the length of ICU or hospital stay. Once again, the risk of hypoglycaemia was significantly higher in the intensively treated group than conventionally treated (6.8% *vs* 0.5%). Thereafter, glycaemic targets in ICU have been pragmatically orientated at 8 - 10 mmol/l (112).

By contrast, there has been little direct evidence that treating hyperglycaemia reduces morbidity or mortality on a general medical or surgical ward. New hyperglycaemia in hospitalized patients, of any aetiology, is associated with a much greater risk of mortality than chronic hyperglycaemia (113). Acute hyperglycaemia affects the innate and adaptive immune responses at multiple levels: it reduces neutrophil degranulation, chemotaxis, and phagocytic activity; impairs complement activation; and inhibits lymphocyte proliferative response (114). However, the pathogenesis of hyperglycaemia is important for the interpretation of clinical outcome data as in those without pre-existing diabetes it has worse prognosis. In these cases, it may be that hyperglycaemia is a surrogate for illness severity.

Historically, the effect of hyperglycaemia on viral outcomes has been less clear (44). However, given the unique interplay between hyperglycaemia and SARS-CoV-2 replication, an *a priori* case can be made for glycaemic control to reduce the severity of COVID-19. Retrospective reports have shown that glucose control preceding admission impacts illness severity and mortality (27, 29). Few data exist for post-admission glycaemic control. In a small study of 25 patients with hyperglycaemia and hospitalised with COVID-19, use of intravenous insulin to achieve a mean glucose of 7.69 ± 1.85 mmol/L (*vs* 10.65 ± 0.84 mmol/L in the no insulin infusion group) was associated with reduced IL-6 and D-dimer levels and improved composite end-point (admission to an ICU, the use of mechanical ventilation, or death) (23).

### Anti-Catabolic Action

#### Hepatic Glucose Production and Peripheral Glucose Uptake

Glucose infusion at 4mg/kg/min, raising blood glucose to 10mmol/L and endogenous plasma insulin to ~400pmol/L failed to suppress lipolysis following elective colorectal surgery (115). By contrast, normalisation of blood glucose (to 5.9 ± 0.3 mmol/L) with exogenous insulin can significantly reduce plasma triglycerides within 24 hours (116), through suppression of lipolysis (68). Therefore, infusion of glucose, without concomitant insulin, is unable to suppress lipolysis in critical illness.

Normalisation of blood glucose is associated with an increase of peripheral glucose uptake (68, 116), but it has been suggested that exogenous insulin administration is unable to overcome hepatic glucose production in critically-ill patients (117). Insulin regulates hepatic gluconeogenesis *via* phosphoenolpyruvate carboxylase (PEPCK) which decarboxylates oxaloacetate to phosphoenolpyruvate in the gluconeogenic pathway. Uncontrolled expression of PEPCK was associated with poor prognosis in critically-ill patients (117), which led the authors to conclude that hepatic insulin resistance could not be overcome and that normalisation of blood glucose with insulin in critically-ill patients must instead be attributable to increasing glucose disposal. However, these data came from post-mortem studies and so the lack of an hepatic effect of insulin might simply represent the degree of metabolic derangement associated with illness severity: for instance glucocorticoids can independently up-regulate PEPCK gene expression (97). Patients in this study had an ICU stay greater than 5 days. This is pertinent as it has been proposed that the site of insulin-resistance could change with time; within 24 hours postoperatively it is mainly the peripheral tissues that are affected (118), whereas by the third postoperative day, the liver appears to be most resistant to insulin (119). Our group has shown that variable dose intravenous insulin administered to medical ICU patients for 48 hours (started within 36 hours of admission), to maintain blood glucose between 7 – 9 mmol/l is sufficient to limit hepatic glucose production rate (68).

### Protein Turnover

Glucose intolerance seen in critical-illness is but one manifestation of insulin resistance – a process that could also manifest in terms of muscle protein catabolism.

Insulin’s effect on protein metabolism in the healthy adult has been contentious but it appears primarily to act *via* the inhibition of proteolysis (120–122), although increased protein synthesis has also been suggested (123). Interpreting the mechanism of action of insulin on protein anabolism is complicated by its other physiological action – that of causing hypoaminoacidaemia. Models of protein turnover involving the measurement of blood-flow across a limb combined with muscle biopsies have been used, predominantly in burned subjects, to examine the effect of insulin on protein turnover. It has been considered that critical-illness leads to impaired amino acid uptake by myocytes, resulting in reduced protein synthesis. In two papers it was suggested that resistance to amino acid uptake may be overcome by a combination of high-dose insulin infusion (to achieve plasma insulin concentrations in the range of 2000 to 5000 pmol/L) plus amino acid provision (124, 125). This had the effect of increasing protein synthesis by approximately 350%, due to a six-fold increase in amino acid transport into the cells. As amino acids by themselves were unable to fully support protein synthesis, it was suggested that insulin may have an independent role in protein synthesis. However, two groups have used the amino acid clamp technique to show that in the presence of adequate amino acid availability, increasing the insulin concentration had no further effect on protein synthesis (126, 127). One small study showed decreased whole-body protein breakdown and synthesis in cardiac surgery patients when administering glucose and insulin under maintenance of normoglycemia (128). However, other studies of ICU patients randomized to tight blood glucose control (4.4-6.1 mmol/L) with conventional, low-dose, insulin infusion protocols have shown no amelioration of muscle loss (81, 129), or whole-body protein turnover (68). None of these studies delivered supplemental amino acids although 0.13 to 0.26 g of nitrogen per kilogram per 24 hours was the standard approach, within 24 hours of ICU admission (68, 81).

What if a hyperinsulinaemic approach was used, rather than conventional low-dose insulin? Exogenous provision of glucose has several theoretical benefits in terms of protein sparing. Firstly, it would be expected to shift substrate utilisation towards increased oxidation of glucose instead of protein. Secondly, exogenous glucose might decrease hepatic glucose production and thereby act indirectly to reduce the need for gluconeogenic precursors. Thirdly, it might drive the accompanying need for insulin and the benefits on protein sparing that might ensue. We have previously reported that despite the infusion of high-dose insulin, causing a six-fold rise in plasma insulin (to ~1500 pmol/L) over the conventional insulin infusion rate, proteolysis was unaffected and remained significantly higher than in the control subjects (68). Such a finding is consistent with previous observations, in both normal subjects and surgical patients, that glucose administration (≥ 4mg/kg/min, causing a doubling of insulin concentration) does not influence the degradation of peripheral protein (130, 131). Our group has also shown that insulin and glucose administration was capable of full suppression of glucose rate of appearance despite ongoing proteolysis (68), suggesting that the function of proteolysis is not to provide gluconeogenic precursors.

## Future Research Questions

Work is needed to further understand the interplay between diabetes and COVID-19. Mechanistic studies are needed to determine the effect of COVID-19 on tissue-specific insulin resistance, the impact on pancreatic B-cell dysfunction, and pulmonary perfusion in the presence of hyperglycaemia (44, 45). The international CoviDiab registry is expected to address a number of these questions (20, 44). Knowledge by which SARS-CoV-2 impacts upon glucose metabolism will be critical for understanding disease pathogenesis and development or choice of therapies.

It would be unrealistic to expect a prospective randomised controlled trial of glucose normalisation on COVID-19 outcomes, but effort must be made for retrospective analyses of propensity-matched subjects. Attention must also be paid to the long-term metabolic sequalae of COVID – given the catabolic processes outlined in this review. Further data are needed on COVID-19 survivors for nutritional status and measures of functional independence in the months after critical care for COVID-19. Early rehabilitation programs are already being evaluated in ongoing clinical studies (132).

## Conclusion

Following the RECOVERY trial results, the use of short-courses of glucocorticoid therapy will be widespread in the remaining time of the COVID-19 pandemic. Based upon the evidence reviewed, the ten-day course of the RECOVERY protocol will be expected to increase both hepatic and peripheral insulin resistance and lead to skeletal muscle loss. Current evidence suggests exogenous insulin should be able to overcome the hepatic and peripheral insulin resistance of glucose metabolism but is unlikely to impact upon skeletal muscle loss engendered by glucocorticoids. Strategies to achieve glycaemic normalisation might have a direct disease modifying effect on the SARS-CoV-2 virus. Further work is needed to develop strategies to limit muscle loss. Even so, we may see a long-term effect on functional capacity from the critical-illness induced by COVID-19.

## Author Contributions

All authors contributed to the article and approved the submitted version.

## Conflict of Interest

The authors declare that the research was conducted in the absence of any commercial or financial relationships that could be construed as a potential conflict of interest.

## References

[1] Horby P Lim WS Emberson JR Mafham M Bell JL Linsell L. Recovery Collaborative Group. Dexamethasone in Hospitalized Patients With Covid-19 - Preliminary Report. N Engl J Med (2021) 384(8):693–704. 10.1101/2020.06.22.20137273 32678530 PMC7383595

[2] Waljee AK Rogers MA Lin P Singal AG Stein JD Marks RM. Short Term Use of Oral Corticosteroids and Related Harms Among Adults in the United States: Population Based Cohort Study. BMJ (2017) 357:j1415. 10.1136/bmj.j1415 28404617 PMC6284230

[3] Villar J Ferrando C Martinez D Ambros A Munoz T Soler JA. Dexamethasone Treatment for the Acute Respiratory Distress Syndrome: A Multicentre, Randomised Controlled Trial. Lancet Respir Med (2020) 8:267–76. 10.1016/S2213-2600(19)30417-5 32043986

[4] Zayed Y Barbarawi M Ismail E Samji V Kerbage J Rizk F. Use of Glucocorticoids in Patients With Acute Respiratory Distress Syndrome: A Meta-Analysis and Trial Sequential Analysis. J Intensive Care (2020) 8:43. 10.1186/s40560-020-00464-1 32612838 PMC7324774

[5] Singh AK Majumdar S Singh R Misra A. Role of Corticosteroid in the Management of COVID-19: A Systemic Review and a Clinician’s Perspective. Diabetes Metab Syndr (2020) 14:971–8. 10.1016/j.dsx.2020.06.054 PMC7320713 32610262

[6] Rizk JG Kalantar-Zadeh K Mehra MR Lavie CJ Rizk Y Forthal DN. Pharmaco-Immunomodulatory Therapy in COVID-19. Drugs (2020) 80:1267–92. 10.1007/s40265-020-01367-z PMC7372203 32696108

[7] Spoorenberg SM Deneer VH Grutters JC Pulles AE Voorn GP Rijkers GT. Pharmacokinetics of Oral vs. Intravenous Dexamethasone in Patients Hospitalized With Community-Acquired Pneumonia. Br J Clin Pharmacol (2014) 78:78–83. 10.1111/bcp.12295 24400953 PMC4168382

[8] Sterne JAC Murthy S Diaz JV Slutsky AS Villar J Angus DC. W. H. O. Rapid Evidence Appraisal for COVID-19 Therapies Working Group. Association Between Administration of Systemic Corticosteroids and Mortality Among Critically Ill Patients With COVID-19: A Meta-Analysis. JAMA (2020) 324:1330–41. 10.1001/jama.2020.17023 PMC7489434 32876694

[9] Jeronimo CMP Farias MEL Val FFA Sampaio VS Alexandre MAA Melo GC. Methylprednisolone as Adjunctive Therapy for Patients Hospitalized With COVID-19 (Metcovid): A Randomised, Double-Blind, Phase IIb, Placebo-Controlled Trial. Clin Infect Dis (2020) 72(9):e373–81. 10.1093/cid/ciaa1177 PMC7454320 32785710

[10] European Medicines Agency. Dexamethasone in Hospitalised Patients With COVID-19. (2020) European Medicine Agency, EMEA/H/A-5(3)/1500.

[11] Abbas N Elhassan M Kelly P Yorke R Mustafa OG Whyte MB. Greater Illness Severity Characterises Steroid Diabetes Following Acute Hospitalisation. Clin Med (Lond) (2019) 19:86–7. 10.7861/clinmedicine.19-1-86 PMC6399644 30651256

[12] Burt MG Roberts GW Aguilar-Loza NR Frith P Stranks SN. Continuous Monitoring of Circadian Glycemic Patterns in Patients Receiving Prednisolone for COPD. J Clin Endocrinol Metab (2011) 96:1789–96. 10.1210/jc.2010-2729 21411550

[13] Fong AC Cheung NW. The High Incidence of Steroid-Induced Hyperglycaemia in Hospital. Diabetes Res Clin Pract (2013) 99:277–80. 10.1016/j.diabres.2012.12.023 23298665

[14] Katsuyama T Sada KE Namba S Watanabe H Katsuyama E Yamanari T. Risk Factors for the Development of Glucocorticoid-Induced Diabetes Mellitus. Diabetes Res Clin Pract (2015) 108:273–9. 10.1016/j.diabres.2015.02.010 25765669

[15] Feo CV Sortini D Ragazzi R De Palma M Liboni A. Randomized Clinical Trial of the Effect of Preoperative Dexamethasone on Nausea and Vomiting After Laparoscopic Cholecystectomy. Br J Surg (2006) 93:295–9. 10.1002/bjs.5252 16400707

[16] Feroci F Rettori M Borrelli A Lenzi E Ottaviano A Scatizzi M. Dexamethasone Prophylaxis Before Thyroidectomy to Reduce Postoperative Nausea, Pain, and Vocal Dysfunction: A Randomized Clinical Controlled Trial. Head Neck (2011) 33:840–6. 10.1002/hed.21543 20737495

[17] Waldron NH Jones CA Gan TJ Allen TK Habib AS. Impact of Perioperative Dexamethasone on Postoperative Analgesia and Side-Effects: Systematic Review and Meta-Analysis. Br J Anaesth (2013) 110:191–200. 10.1093/bja/aes431 23220857 PMC3544008

[18] Pasternak JJ McGregor DG Lanier WL. Effect of Single-Dose Dexamethasone on Blood Glucose Concentration in Patients Undergoing Craniotomy. J Neurosurg Anesthesiol (2004) 16:122–5. 10.1097/00008506-200404000-00003 15021280

[19] Hans P Vanthuyne A Dewandre PY Brichant JF Bonhomme V. Blood Glucose Concentration Profile After 10 mg Dexamethasone in Non-Diabetic and Type 2 Diabetic Patients Undergoing Abdominal Surgery. Br J Anaesth (2006) 97:164–70. 10.1093/bja/ael111 16698859

[20] Rubino F Amiel SA Zimmet P Alberti G Bornstein S Eckel RH. New-Onset Diabetes in Covid-19. N Engl J Med (2020) 383:789–90. 10.1056/NEJMc2018688 PMC7304415 32530585

[21] Guan WJ Ni ZY Hu Y Liang WH Ou CQ He JX. Clinical Characteristics of Coronavirus Disease 2019 in China. N Engl J Med (2020) 382:1708–20. 10.1056/NEJMoa2002032 PMC7092819 32109013

[22] Iacobellis G Penaherrera CA Bermudez LE Bernal Mizrachi E. Admission Hyperglycemia and Radiological Findings of SARS-CoV2 in Patients With and Without Diabetes. Diabetes Res Clin Pract (2020) 164:108185. 10.1016/j.diabres.2020.108185 32360710 PMC7251996

[23] Sardu C D’Onofrio N Balestrieri ML Barbieri M Rizzo MR Messina V. Outcomes in Patients With Hyperglycemia Affected by COVID-19: Can We Do More on Glycemic Control? Diabetes Care (2020) 43:1408–15. 10.2337/dc20-0723 PMC7305003 32430456

[24] Vargas-Vazquez A Bello-Chavolla OY Ortiz-Brizuela E Campos-Munoz A Mehta R Villanueva-Reza M. Impact of Undiagnosed Type 2 Diabetes and Pre-Diabetes on Severity and Mortality for SARS-CoV-2 Infection. BMJ Open Diabetes Res Care (2021) 9. 10.1136/bmjdrc-2020-002026 PMC7887863 33593750

[25] Wu J Huang J Zhu G Wang Q Lv Q Huang Y. Elevation of Blood Glucose Level Predicts Worse Outcomes in Hospitalized Patients With COVID-19: A Retrospective Cohort Study. BMJ Open Diabetes Res Care (2020) 8. 10.1136/bmjdrc-2020-001476 PMC7298690 32503812

[26] Zhang J Kong W Xia P Xu Y Li L Li Q. Impaired Fasting Glucose and Diabetes are Related to Higher Risks of Complications and Mortality Among Patients With Coronavirus Disease 2019. Front Endocrinol (Lausanne) (2020) 11:525. 10.3389/fendo.2020.00525 32754119 PMC7365851

[27] Holman N Knighton P Kar P O’Keefe J Curley M Weaver A. Risk Factors for COVID-19-Related Mortality in People With Type 1 and Type 2 Diabetes in England: A Population-Based Cohort Study. Lancet Diabetes Endocrinol (2020) 8:823–33. 10.1016/S2213-8587(20)30271-0 PMC7426091 32798471

[28] Merzon E Green I Shpigelman M Vinker S Raz I Golan-Cohen A. Haemoglobin A1c is a Predictor of COVID-19 Severity in Patients With Diabetes. Diabetes Metab Res Rev (2020), e3398. 10.1002/dmrr.3398 32852883 PMC7460936

[29] Williamson EJ Walker AJ Bhaskaran K Bacon S Bates C Morton CE. Factors Associated With COVID-19-Related Death Using Opensafely. Nature (2020) 584:430–6. 10.1038/s41586-020-2521-4 PMC7611074 32640463

[30] Yang JK Lin SS Ji XJ Guo LM. Binding of SARS Coronavirus to its Receptor Damages Islets and Causes Acute Diabetes. Acta Diabetol (2010) 47:193–9. 10.1007/s00592-009-0109-4 PMC7088164 19333547

[31] Yang L Han Y Nilsson-Payant BE Gupta V Wang P Duan X. A Human Pluripotent Stem Cell-Based Platform to Study SARS-CoV-2 Tropism and Model Virus Infection in Human Cells and Organoids. Cell Stem Cell (2020) 27:125–36.e7. 10.1016/j.stem.2020.06.015 32579880 PMC7303620

[32] Chee YJ Ng SJH Yeoh E. Diabetic Ketoacidosis Precipitated by Covid-19 in a Patient With Newly Diagnosed Diabetes Mellitus. Diabetes Res Clin Pract (2020) 164:108166. 10.1016/j.diabres.2020.108166 32339533 PMC7194589

[33] Li J Wang X Chen J Zuo X Zhang H Deng A. COVID-19 Infection May Cause Ketosis and Ketoacidosis. Diabetes Obes Metab (2020) 2(10):1935–40. 10.1111/dom.14057 PMC7264681 32314455

[34] Smith SM Boppana A Traupman JA Unson E Maddock DA Chao K. Impaired Glucose Metabolism in Patients With Diabetes, Prediabetes, and Obesity Is Associated With Severe COVID-19. J Med Virol (2021) 93:409–15. 10.1002/jmv.26227 PMC7361926 32589756

[35] Kamrath C Monkemoller K Biester T Rohrer TR Warncke K Hammersen J. Ketoacidosis in Children and Adolescents With Newly Diagnosed Type 1 Diabetes During the COVID-19 Pandemic in Germany. JAMA (2020) 324:801–4. 10.1001/jama.2020.13445 PMC7372511 32702751

[36] Lawrence C Seckold R Smart C King BR Howley P Feltrin R. Increased Paediatric Presentations of Severe Diabetic Ketoacidosis in an Australian Tertiary Centre During the COVID-19 Pandemic. Diabetes Med (2021) 38:e14417. 10.1111/dme.14417 PMC7646057 33020999

[37] Tittel SR Rosenbauer J Kamrath C Ziegler J Reschke F Hammersen J. Did the COVID-19 Lockdown Affect the Incidence of Pediatric Type 1 Diabetes in Germany? Diabetes Care (2020) 43:e172–3. 10.2337/dc20-1633 PMC7576433 32826282

[38] Hippich M Holthaus L Assfalg R Zapardiel-Gonzalo J Kapfelsperger H Heigermoser M. A Public Health Antibody Screening Indicates a 6-Fold Higher SARS-CoV-2 Exposure Rate Than Reported Cases in Children. Med (N Y) (2021) 2:149–63.e4. 10.1016/j.medj.2020.10.003 33163984 PMC7598360

[39] Coate KC Cha J Shrestha S Wang W Goncalves LM Almaca J. SARS-CoV-2 Cell Entry Factors ACE2 and TMPRSS2 Are Expressed in the Microvasculature and Ducts of Human Pancreas But Are Not Enriched in Beta Cells. Cell Metab (2020) 32(6):1028–40.e4. 10.1016/j.cmet.2020.11.006 PMC7664344 33207245

[40] Wang F Wang H Fan J Zhang Y Wang H Zhao Q. Pancreatic Injury Patterns in Patients With Coronavirus Disease 19 Pneumonia. Gastroenterology (2020) 159:367–70. 10.1053/j.gastro.2020.03.055 PMC7118654 32247022

[41] Armeni E Aziz U Qamar S Nasir S Nethaji C Negus R. Protracted Ketonaemia in Hyperglycaemic Emergencies in COVID-19: A Retrospective Case Series. Lancet Diabetes Endocrinol (2020) 8:660–3. 10.1016/S2213-8587(20)30221-7 PMC7329282 32621809

[42] Vellanki P Umpierrez GE. Diabetic Ketoacidosis: A Common Debut of Diabetes Among African Americans With Type 2 Diabetes. Endocr Pract (2017) 23:971–8. 10.4158/EP161679.RA PMC6092188 28534682

[43] Codo AC Davanzo GG Monteiro LB de Souza GF Muraro SP Virgilio-da-Silva JV. Elevated Glucose Levels Favor SARS-CoV-2 Infection and Monocyte Response Through a HIF-1alpha/Glycolysis-Dependent Axis. Cell Metab (2020) 32:498–9. 10.2139/ssrn.3606770 PMC7462530 32877692

[44] Vas P Hopkins D Feher M Rubino F Whyte MB. Diabetes, Obesity and COVID-19: A Complex Interplay. Diabetes Obes Metab (2020) 22:1892–6. 10.1111/dom.14134 PMC7362013 32627299

[45] Whyte MB Vas P Heiss C Feher MD. The Contribution of Diabetic Micro-Angiopathy to Adverse Outcomes in COVID-19. Diabetes Res Clin Pract (2020) 164:108217. 10.1016/j.diabres.2020.108217 32451317 PMC7217793

[46] Nicholson G Burrin JM Hall GM. Peri-Operative Steroid Supplementation. Anaesthesia (1998) 53:1091–104. 10.1046/j.1365-2044.1998.00578.x 10023279

[47] Boonen E Vervenne H Meersseman P Andrew R Mortier L Declercq PE. Reduced Cortisol Metabolism During Critical Illness. N Engl J Med (2013) 368:1477–88. 10.1056/NEJMoa1214969 PMC4413428 23506003

[48] Williams DM. Clinical Pharmacology of Corticosteroids. Respir Care (2018) 63:655–70. 10.4187/respcare.06314 29794202

[49] Mastorakos G Chrousos GP Weber JS. Recombinant Interleukin-6 Activates the Hypothalamic-Pituitary-Adrenal Axis in Humans. J Clin Endocrinol Metab (1993) 77:1690–4. 10.1210/jc.77.6.1690 8263159

[50] Khoo B Boshier PR Freethy A Tharakan G Saeed S Hill N. Redefining the Stress Cortisol Response to Surgery. Clin Endocrinol (Oxf) (2017) 87:451–8. 10.1111/cen.13439 28758231

[51] Meyer EJ Nenke MA Rankin W Lewis JG Konings E Slager M. Total and High-Affinity Corticosteroid-Binding Globulin Depletion in Septic Shock Is Associated With Mortality. Clin Endocrinol (Oxf) (2019) 90:232–40. 10.1111/cen.13844 30160799

[52] Tan T Khoo B Mills EG Phylactou M Patel B Eng PC. Association Between High Serum Total Cortisol Concentrations and Mortality From COVID-19. Lancet Diabetes Endocrinol (2020) 8:659–60. 10.1016/S2213-8587(20)30216-3 PMC7302794 32563278

[53] Gibbison B Keenan DM Roelfsema F Evans J Phillips K Rogers CA. Dynamic Pituitary-Adrenal Interactions in the Critically Ill After Cardiac Surgery. J Clin Endocrinol Metab (2020) 105(5):1327–42. 10.1210/clinem/dgaa422 PMC7089849 31738827

[54] Boonen E Meersseman P Vervenne H Meyfroidt G Guiza F Wouters PJ. Reduced Nocturnal ACTH-Driven Cortisol Secretion During Critical Illness. Am J Physiol Endocrinol Metab (2014) 306:E883–92. 10.1152/ajpendo.00009.2014 PMC3989736 24569590

[55] Teblick A Langouche L Van den Berghe G. Anterior Pituitary Function in Critical Illness. Endocr Connect (2019) 8:R131–43. 10.1530/EC-19-0318 PMC6709544 31340197

[56] Bornstein SR Rutkowski H Vrezas I. Cytokines and Steroidogenesis. Mol Cell Endocrinol (2004) 215:135–41. 10.1016/j.mce.2003.11.022 15026186

[57] Kauh EA Mixson LA Shankar S McCarthy J Maridakis V Morrow L. Short-Term Metabolic Effects of Prednisone Administration in Healthy Subjects. Diabetes Obes Metab (2011) 13:1001–7. 10.1111/j.1463-1326.2011.01432.x 21635675

[58] van Raalte DH Brands M van der Zijl NJ Muskiet MH Pouwels PJ Ackermans MT. Low-Dose Glucocorticoid Treatment Affects Multiple Aspects of Intermediary Metabolism in Healthy Humans: A Randomised Controlled Trial. Diabetologia (2011) 54:2103–12. 10.1007/s00125-011-2174-9 PMC3131514 21562755

[59] Virkamaki A Puhakainen I Koivisto VA Vuorinen-Markkola H Yki-Jarvinen H. Mechanisms of Hepatic and Peripheral Insulin Resistance During Acute Infections in Humans. J Clin Endocrinol Metab (1992) 74:673–9. 10.1210/jcem.74.3.1740504 1740504

[60] Andreis DT Singer M. Catecholamines for Inflammatory Shock: A Jekyll-and-Hyde Conundrum. Intensive Care Med (2016) 42:1387–97. 10.1007/s00134-016-4249-z 26873833

[61] Morigny P Houssier M Mouisel E Langin D. Adipocyte Lipolysis and Insulin Resistance. Biochimie (2016) 125:259–66. 10.1016/j.biochi.2015.10.024 26542285

[62] Thiessen SE Derde S Derese I Dufour T Vega CA Langouche L. Role of Glucagon in Catabolism and Muscle Wasting of Critical Illness and Modulation by Nutrition. Am J Respir Crit Care Med (2017) 196:1131–43. 10.1164/rccm.201702-0354OC 28475354

[63] Zeng Z Yu H Chen H Qi W Chen L Chen G. Longitudinal Changes of Inflammatory Parameters and Their Correlation With Disease Severity and Outcomes in Patients With COVID-19 From Wuhan, China. Crit Care (2020) 24:525. 10.1186/s13054-020-03255-0 32854750 PMC7450961

[64] Lockhart SG Griffiths H Petrisor B Usman A Calvo-Latorre J Heales L. The Excess Insulin Requirement in Severe COVID-19 Compared to Non-COVID-19 Viral Pneumonitis Is Related to the Severity of Respiratory Failure and Pre-Existing Diabetes. Crit Care (2020) 24(1):525. 10.1101/2020.10.03.20206284 34268452 PMC7995054

[65] Zauner A Nimmerrichter P Anderwald C Bischof M Schiefermeier M Ratheiser K. Severity of Insulin Resistance in Critically Ill Medical Patients. Metabolism (2007) 56:1–5. 10.1016/j.metabol.2006.08.014 17161218

[66] Revelly JP Tappy L Martinez A Bollmann M Cayeux MC Berger MM. Lactate and Glucose Metabolism in Severe Sepsis and Cardiogenic Shock. Crit Care Med (2005) 33:2235–40. 10.1097/01.CCM.0000181525.99295.8F 16215376

[67] Shaw JH Klein S Wolfe RR. Assessment of Alanine, Urea, and Glucose Interrelationships in Normal Subjects and in Patients With Sepsis With Stable Isotopic Tracers. Surgery (1985) 97:557–68. 10.1016/j.cmi.2020.10.021 3887629

[68] Whyte MB Jackson NC Shojaee-Moradie F Treacher DF Beale RJ Jones RH. Metabolic Effects of Intensive Insulin Therapy in Critically Ill Patients. Am J Physiol Endocrinol Metab (2010) 298:E697–705. 10.1152/ajpendo.00407.2009 20028969

[69] Ferenci T Benyo B Kovacs L Fisk L Shaw GM Chase JG. Daily Evolution of Insulin Sensitivity Variability With Respect to Diagnosis in the Critically Ill. PloS One (2013) 8:e57119. 10.1371/journal.pone.0057119 23437328 PMC3578812

[70] Pretty CG Le Compte AJ Chase JG Shaw GM Preiser JC Penning S. Variability of Insulin Sensitivity During the First 4 Days of Critical Illness: Implications for Tight Glycemic Control. Ann Intensive Care (2012) 2:17. 10.1186/2110-5820-2-17 22703645 PMC3464183

[71] Yki-Jarvinen H Sammalkorpi K Koivisto VA Nikkila EA. Severity, Duration, and Mechanisms of Insulin Resistance During Acute Infections. J Clin Endocrinol Metab (1989) 69:317–23. 10.1210/jcem-69-2-317 2666428

[72] Marik PE Bellomo R. Stress Hyperglycemia: An Essential Survival Response! Crit Care (2013) 17:305. 10.1186/cc12514 23470218 PMC3672537

[73] Wandrag L Brett SJ Frost GS Bountziouka V Hickson M. Exploration of Muscle Loss and Metabolic State During Prolonged Critical Illness: Implications for Intervention? PloS One (2019) 14:e0224565. 10.1371/journal.pone.0224565 31725748 PMC6855435

[74] Barberis E Timo S Amede E Vanella VV Puricelli C Cappellano G. Large-Scale Plasma Analysis Revealed New Mechanisms and Molecules Associated With the Host Response to SARS-CoV-2. Int J Mol Sci (2020) 21(22):8623. 10.3390/ijms21228623 PMC7696386 33207699

[75] Rittig N Bach E Thomsen HH Pedersen SB Nielsen TS Jorgensen JO. Regulation of Lipolysis and Adipose Tissue Signaling During Acute Endotoxin-Induced Inflammation: A Human Randomized Crossover Trial. PloS One (2016) 11:e0162167. 10.1371/journal.pone.0162167 27627109 PMC5023116

[76] Ilias I Vassiliadi DA Theodorakopoulou M Boutati E Maratou E Mitrou P. Adipose Tissue Lipolysis and Circulating Lipids in Acute and Subacute Critical Illness: Effects of Shock and Treatment. J Crit Care (2014) 29:1130 e5–9. 10.1016/j.jcrc.2014.06.003 25012960

[77] Wolfe RR Shaw JH Durkot MJ. Effect of Sepsis on VLDL Kinetics: Responses in Basal State and During Glucose Infusion. Am J Physiol (1985) 248:E732–40. 10.1152/ajpendo.1985.248.6.E732 3890559

[78] Ali Abdelhamid Y Cousins CE Sim JA Bellon MS Nguyen NQ Horowitz M. Effect of Critical Illness on Triglyceride Absorption. JPEN J Parenter Enteral Nutr (2015) 39:966–72. 10.1177/0148607114540214 24963026

[79] Dusseaux MM Antoun S Grigioni S Beduneau G Carpentier D Girault C. Skeletal Muscle Mass and Adipose Tissue Alteration in Critically Ill Patients. PloS One (2019) 14:e0216991. 10.1371/journal.pone.0216991 31194755 PMC6563951

[80] Wollersheim T Woehlecke J Krebs M Hamati J Lodka D Luther-Schroeder A. Dynamics of Myosin Degradation in Intensive Care Unit-Acquired Weakness During Severe Critical Illness. Intensive Care Med (2014) 40:528–38. 10.1007/s00134-014-3224-9 24531339

[81] Derde S Hermans G Derese I Guiza F Hedstrom Y Wouters PJ. Muscle Atrophy and Preferential Loss of Myosin in Prolonged Critically Ill Patients. Crit Care Med (2012) 40:79–89. 10.1097/CCM.0b013e31822d7c18 21926599

[82] Puthucheary ZA Rawal J McPhail M Connolly B Ratnayake G Chan P. Acute Skeletal Muscle Wasting in Critical Illness. JAMA (2013) 310:1591–600. 10.1001/jama.2013.278481 24108501

[83] Vanhorebeek I Latronico N Van den Berghe G. ICU-Acquired Weakness. Intensive Care Med (2020) 46:637–53. 10.1007/s00134-020-05944-4 PMC7224132 32076765

[84] Arnold J Campbell IT Samuels TA Devlin JC Green CJ Hipkin LJ. Increased Whole Body Protein Breakdown Predominates Over Increased Whole Body Protein Synthesis in Multiple Organ Failure. Clin Sci (Lond) (1993) 84:655–61. 10.1042/cs0840655 8334812

[85] Berg A Rooyackers O Bellander BM Wernerman J. Whole Body Protein Kinetics During Hypocaloric and Normocaloric Feeding in Critically Ill Patients. Crit Care (2013) 17:R158. 10.1186/cc12837 23883571 PMC4057244

[86] Darmaun D Matthews DE Bier DM. Physiological Hypercortisolemia Increases Proteolysis, Glutamine, and Alanine Production. Am J Physiol (1988) 255:E366–73. 10.1152/ajpendo.1988.255.3.E366 3048115

[87] Flakoll PJ Hill JO Abumrad NN. Acute Hyperglycemia Enhances Proteolysis in Normal Man. Am J Physiol (1993) 265:E715–21. 10.1152/ajpendo.1993.265.5.E715 8238497

[88] Gore DC Chinkes DL Hart DW Wolf SE Herndon DN Sanford AP. Hyperglycemia Exacerbates Muscle Protein Catabolism in Burn-Injured Patients. Crit Care Med (2002) 30:2438–42. 10.1097/00003246-200211000-00006 12441751

[89] Friedrich O Reid MB Van den Berghe G Vanhorebeek I Hermans G Rich MM. The Sick and the Weak: Neuropathies/Myopathies in the Critically Ill. Physiol Rev (2015) 95:1025–109. 10.1152/physrev.00028.2014 PMC4491544 26133937

[90] Lightfoot A McArdle A Griffiths RD. Muscle in Defense. Crit Care Med (2009) 37:S384–90. 10.1097/CCM.0b013e3181b6f8a5 20046124

[91] Jackson NC Carroll PV Russell-Jones DL Sonksen PH Treacher DF Umpleby AM. The Metabolic Consequences of Critical Illness: Acute Effects on Glutamine and Protein Metabolism. Am J Physiol (1999) 276:E163–70. 10.1152/ajpendo.1999.276.1.E163 9886963

[92] Cruzat V Macedo Rogero M Noel Keane K Curi R Newsholme P. Glutamine: Metabolism and Immune Function, Supplementation and Clinical Translation. Nutrients (2018) 10(11):1564. 10.20944/preprints201809.0459.v1 PMC6266414 30360490

[93] Ripa M Galli L Poli A Oltolini C Spagnuolo V Mastrangelo A. Secondary Infections in Patients Hospitalized With COVID-19: Incidence and Predictive Factors. Clin Microbiol Infect (2020) 27(3):451–7. 10.1016/j.cmi.2020.10.021 PMC7584496 33223114

[94] European Centre for Disease Prevention and Control. Incidence and Attributable Mortality of Healthcare-Associated Infections in Intensive Care Units in Europe, 2008–2012. Stockholm: European Centre for Disease Prevention and Control (2018).

[95] Langford BJ So M Raybardhan S Leung V Westwood D MacFadden DR. Bacterial Co-Infection and Secondary Infection in Patients With COVID-19: A Living Rapid Review and Meta-Analysis. Clin Microbiol Infect (2020) 26:1622–9. 10.1016/j.cmi.2020.07.016 PMC7832079 32711058

[96] Alessi J de Oliveira GB Schaan BD Telo GH. Dexamethasone in the Era of COVID-19: Friend or Foe? An Essay on the Effects of Dexamethasone and the Potential Risks of its Inadvertent Use in Patients With Diabetes. Diabetol Metab Syndr (2020) 12:80. 10.1186/s13098-020-00583-7 32922517 PMC7476640

[97] Sasaki K Cripe TP Koch SR Andreone TL Petersen DD Beale EG. Multihormonal Regulation of Phosphoenolpyruvate Carboxykinase Gene Transcription. The Dominant Role of Insulin. J Biol Chem (1984) 259:15242–51. 10.1016/S0021-9258(17)42541-5 6096365

[98] Kuo T McQueen A Chen TC Wang JC. Regulation of Glucose Homeostasis by Glucocorticoids. Adv Exp Med Biol (2015) 872:99–126. 10.1007/978-1-4939-2895-8_5 26215992 PMC6185996

[99] Eriksen M Jensen DH Tribler S Holst JJ Madsbad S Krarup T. Reduction of Insulinotropic Properties of GLP-1 and GIP After Glucocorticoid-Induced Insulin Resistance. Diabetologia (2015) 58:920–8. 10.1007/s00125-015-3522-y 25748606

[100] Petersons CJ Mangelsdorf BL Jenkins AB Poljak A Smith MD Greenfield JR. Effects of Low-Dose Prednisolone on Hepatic and Peripheral Insulin Sensitivity, Insulin Secretion, and Abdominal Adiposity in Patients With Inflammatory Rheumatologic Disease. Diabetes Care (2013) 36:2822–9. 10.2337/dc12-2617 PMC3747874 23670996

[101] Braun TP Marks DL. The Regulation of Muscle Mass by Endogenous Glucocorticoids. Front Physiol (2015) 6:12. 10.3389/fphys.2015.00012 25691871 PMC4315033

[102] Burt MG Johannsson G Umpleby AM Chisholm DJ Ho KK. Impact of Acute and Chronic Low-Dose Glucocorticoids on Protein Metabolism. J Clin Endocrinol Metab (2007) 92:3923–9. 10.1210/jc.2007-0951 17652216

[103] Beaufrere B Horber FF Schwenk WF Marsh HM Matthews D Gerich JE. Glucocorticosteroids Increase Leucine Oxidation and Impair Leucine Balance in Humans. Am J Physiol (1989) 257:E712–21. 10.1152/ajpendo.1989.257.5.E712 2596599

[104] Van den Berghe G Wilmer A Hermans G Meersseman W Wouters PJ Milants I. Intensive Insulin Therapy in the Medical ICU. N Engl J Med (2006) 354:449–61. 10.1056/NEJMoa052521 16452557

[105] van den Berghe G Wouters P Weekers F Verwaest C Bruyninckx F Schetz M. Intensive Insulin Therapy in Critically Ill Patients. N Engl J Med (2001) 345:1359–67. 10.1056/NEJMoa011300 11794168

[106] Diaz R Paolasso EA Piegas LS Tajer CD Moreno MG Corvalan R. Metabolic Modulation of Acute Myocardial Infarction. The ECLA (Estudios Cardiologicos Latinoamerica) Collaborative Group. Circulation (1998) 98:2227–34. 10.1161/01.CIR.98.21.2227 9867443

[107] Mehta SR Yusuf S Diaz R Zhu J Pais P Xavier D. Effect of Glucose-Insulin-Potassium Infusion on Mortality in Patients With Acute ST-Segment Elevation Myocardial Infarction: The CREATE-ECLA Randomized Controlled Trial. JAMA (2005) 293:437–46. 10.1001/jama.293.4.437 15671428

[108] Van den Berghe G Wouters PJ Bouillon R Weekers F Verwaest C Schetz M. Outcome Benefit of Intensive Insulin Therapy in the Critically Ill: Insulin Dose Versus Glycemic Control. Crit Care Med (2003) 31:359–66. 10.1097/01.CCM.0000045568.12881.10 12576937

[109] Brunkhorst FM Engel C Bloos F Meier-Hellmann A Ragaller M Weiler N. Intensive Insulin Therapy and Pentastarch Resuscitation in Severe Sepsis. N Engl J Med (2008) 358:125–39. 10.1056/NEJMoa070716 18184958

[110] Preiser JC Devos P Ruiz-Santana S Melot C Annane D Groeneveld J. A Prospective Randomised Multi-Centre Controlled Trial on Tight Glucose Control by Intensive Insulin Therapy in Adult Intensive Care Units: The Glucontrol Study. Intensive Care Med (2009) 35:1738–48. 10.1007/s00134-009-1585-2 19636533

[111] Finfer S Chittock DR Su SY Blair D Foster D Dhingra V. Nice-Sugar Study Investigators. Intensive Versus Conventional Glucose Control in Critically Ill Patients. N Engl J Med (2009) 360:1283–97. 10.1056/NEJMoa0810625 19318384

[112] Bellomo R. Acute Glycemic Control in Diabetics. How Sweet is Oprimal? Pro: Sweeter Is Better in Diabetes. J Intensive Care (2018) 6:71. 10.1186/s40560-018-0336-2 30455957 PMC6225577

[113] Umpierrez GE Isaacs SD Bazargan N You X Thaler LM Kitabchi AE. Hyperglycemia: An Independent Marker of in-Hospital Mortality in Patients With Undiagnosed Diabetes. J Clin Endocrinol Metab (2002) 87:978–82. 10.1210/jcem.87.3.8341 11889147

[114] Jafar N Edriss H Nugent K. The Effect of Short-Term Hyperglycemia on the Innate Immune System. Am J Med Sci (2016) 351:201–11. 10.1016/j.amjms.2015.11.011 26897277

[115] Schricker T Carli F Lattermann R Wachter U Georgieff M. Glucose Infusion Does Not Suppress Increased Lipolysis After Abdominal Surgery. Nutrition (2001) 17:85–90. 10.1016/S0899-9007(00)00491-3 11240333

[116] Mesotten D Swinnen JV Vanderhoydonc F Wouters PJ Van den Berghe G. Contribution of Circulating Lipids to the Improved Outcome of Critical Illness by Glycemic Control With Intensive Insulin Therapy. J Clin Endocrinol Metab (2004) 89:219–26. 10.1210/jc.2003-030760 14715853

[117] Mesotten D Delhanty PJ Vanderhoydonc F Hardman KV Weekers F Baxter RC. Regulation of Insulin-Like Growth Factor Binding Protein-1 During Protracted Critical Illness. J Clin Endocrinol Metab (2002) 87:5516–23. 10.1210/jc.2002-020664 12466347

[118] Nygren J Thorell A Efendic S Nair KS Ljungqvist O. Site of Insulin Resistance After Surgery: The Contribution of Hypocaloric Nutrition and Bed Rest. Clin Sci (Lond) (1997) 93:137–46. 10.1042/cs0930137 9301428

[119] Soop M Nygren J Thorell A Weidenhielm L Lundberg M Hammarqvist F. Preoperative Oral Carbohydrate Treatment Attenuates Endogenous Glucose Release 3 Days After Surgery. Clin Nutr (2004) 23:733–41. 10.1016/j.clnu.2003.12.007 15297112

[120] Castellino P Luzi L Simonson DC Haymond M DeFronzo RA. Effect of Insulin and Plasma Amino Acid Concentrations on Leucine Metabolism in Man. Role of Substrate Availability on Estimates of Whole Body Protein Synthesis. J Clin Invest (1987) 80:1784–93. 10.1172/JCI113272 PMC442454 3316280

[121] Fukagawa NK Minaker KL Rowe JW Goodman MN Matthews DE Bier DM. Insulin-Mediated Reduction of Whole Body Protein Breakdown. Dose-Response Effects on Leucine Metabolism in Postabsorptive Men. J Clin Invest (1985) 76:2306–11. 10.1172/JCI112240 PMC424354 3908486

[122] Tessari P Trevisan R Inchiostro S Biolo G Nosadini R De Kreutzenberg SV. Dose-Response Curves of Effects of Insulin on Leucine Kinetics in Humans. Am J Physiol (1986) 251:E334–42. 10.1152/ajpendo.1986.251.3.E334 3529984

[123] Biolo G Declan Fleming RY Wolfe RR. Physiologic Hyperinsulinemia Stimulates Protein Synthesis and Enhances Transport of Selected Amino Acids in Human Skeletal Muscle. J Clin Invest (1995) 95:811–9. 10.1172/JCI117731 PMC295560 7860765

[124] Ferrando AA Chinkes DL Wolf SE Matin S Herndon DN Wolfe RR. A Submaximal Dose of Insulin Promotes Net Skeletal Muscle Protein Synthesis in Patients With Severe Burns. Ann Surg (1999) 229:11–8. 10.1097/00000658-199901000-00002 PMC1191603 9923795

[125] Sakurai Y Aarsland A Herndon DN Chinkes DL Pierre E Nguyen TT. Stimulation of Muscle Protein Synthesis by Long-Term Insulin Infusion in Severely Burned Patients. Ann Surg (1995) 222:283–94; 294-7. 10.1097/00000658-199509000-00007 7677459 PMC1234807

[126] Flakoll PJ Kulaylat M Frexes-Steed M Hourani H Brown LL Hill JO. Amino Acids Augment Insulin’s Suppression of Whole Body Proteolysis. Am J Physiol (1989) 257:E839–47. 10.1152/ajpendo.1989.257.6.E839 2692456

[127] Russell-Jones DL Umpleby AM Hennessy TR Bowes SB Shojaee-Moradie F Hopkins KD. Use of a Leucine Clamp to Demonstrate That IGF-I Actively Stimulates Protein Synthesis in Normal Humans. Am J Physiol (1994) 267:E591–8. 10.1152/ajpendo.1994.267.4.E591 7943309

[128] Hatzakorzian R Shum-Tim D Wykes L Hulshoff A Bui H Nitschmann E. Glucose and Insulin Administration While Maintaining Normoglycemia Inhibits Whole Body Protein Breakdown and Synthesis After Cardiac Surgery. J Appl Physiol (1985) (2014) 117:1380–7. 10.1152/japplphysiol.00175.2014 25257875

[129] Fisher JG Sparks EA Khan FA Alexander JL Asaro LA Wypij D. Tight Glycemic Control With Insulin Does Not Affect Skeletal Muscle Degradation During the Early Postoperative Period Following Pediatric Cardiac Surgery. Pediatr Crit Care Med (2015) 16:515–21. 10.1097/PCC.0000000000000413 PMC4497866 25850865

[130] Harrison RA Lewin MR Halliday D Clark CG. Leucine Kinetics in Surgical Patients. I: A Study of the Effect of Surgical ‘Stress’. Br J Surg (1989) 76:505–8. 10.1002/bjs.1800760524 2736365

[131] Schricker T Wykes L Carli F. Epidural Blockade Improves Substrate Utilization After Surgery. Am J Physiol Endocrinol Metab (2000) 279:E646–53. 10.1152/ajpendo.2000.279.3.E646 10950834

[132] Nalbandian A Sehgal K Gupta A Madhavan MV McGroder C Stevens JS. Post-Acute COVID-19 Syndrome. Nat Med (2021) 27:601–15. 10.1038/s41591-021-01283-z PMC8893149 33753937

